# A Scoping Review of Microsimulation Models on Obesity-Related Policy Evaluation

**DOI:** 10.3390/nu18010073

**Published:** 2025-12-25

**Authors:** Zhixin Cao, Yue Fang, Chenyu Wang, Ruopeng An

**Affiliations:** 1 Department of Public Health Sciences, University of Chicago, Chicago, IL 60637, USA; 2 Silver School of Social Work, New York University, New York, NY 10012, USA; yf2938@nyu.edu; 3 School of Nursing, University of Michigan, Ann Arbor, MI 48105, USA; cywangg@umich.edu; 4 Constance and Martin Silver Center on Data Science and Social Equity, Silver School of Social Work, New York University, New York, NY 10012, USA; ra4605@nyu.edu

**Keywords:** microsimulation, nutrition policy, food systems, obesity, cost-effectiveness

## Abstract

**Background/Objectives**: Obesity is a major global public health and economic challenge. Governments worldwide have implemented nutrition-focused policies such as sugar-sweetened beverage taxes, front-of-pack labeling, food assistance reforms, and school nutrition standards to improve diet quality and reduce obesity. Because large-scale randomized controlled trials are often infeasible and conventional epidemiologic methods overlook population heterogeneity and behavioral feedback, microsimulation modeling has become a key tool for evaluating long-term and distributional policy impacts. This scoping review examined the application of microsimulation to obesity-related nutrition policies, focusing on model structure, behavioral parameterization, and integration of economic and equity analyses. **Methods**: Following PRISMA guidelines (PROSPERO CRD42024599769), five databases were searched for peer-reviewed studies. Data were extracted on policy mechanisms, model design, parameterization, and equity analysis. Study quality was assessed using a customized 21-item checklist adapted from CHEERS and NIH tools. **Results**: Twenty-nine studies met the inclusion criteria, with most policy settings based in the United States. Most employed dynamic, stochastic, individual-level microsimulation models with diverse behavioral assumptions, obesity equations, and calibration approaches. While most studies stratified outcomes by socioeconomic or demographic group, only one used a formal quantitative equity metric. **Conclusions**: Microsimulation modeling provides valuable evidence on the long-term health, economic, and distributional impacts of nutrition policies. Future work should strengthen methodological transparency, standardize equity assessment, and expand application beyond high-income settings to improve the comparability, credibility, and policy relevance of simulation-based nutrition policy research.

## 1. Introduction

Obesity has become one of the most pressing global nutrition and public health challenges of the 21st century. As of 2022, an estimated 2.5 billion adults worldwide were overweight, including approximately 890 million living with obesity, accounting for nearly 16 percent of the global adult population [1]. Among children and adolescents aged 5 to 19 years, the global prevalence of obesity has quadrupled since 1990 to 8%. In the U.S., nationally representative data for August 2021 to August 2023 show that 40.3% of adults had obesity, continuing a long-term upward trajectory [2]. If the current trends persist, the combined global economic burden of overweight and obesity is projected to exceed USD 3 trillion annually by 2030 and rise beyond USD 18 trillion by 2060, equivalent to approximately 3% of global gross domestic product (GDP) [1,3]. Beyond its clinical implications, obesity imposes sustained fiscal, productivity, and social challenges, highlighting the urgent need for coordinated, nutrition-focused policy responses.

Over the past decade, governments have implemented a range of nutrition-focused policies to enhance dietary quality and prevent obesity, including sugar-sweetened beverage (SSB) taxes, front-of-pack labeling, reformulation targets for salt, sugar, and fat, as well as nutrition standards in schools and public institutions [4,5]. These interventions are grounded in nutritional epidemiology and behavioral nutrition science, aiming to reshape food environments and encourage healthier food choices. Yet, assessing their long-term health and economic impacts remains challenging. Large-scale randomized controlled trials (RCTs), although the gold standard for causal inference, are rarely feasible, ethical, or affordable at the population level. Traditional epidemiologic and econometric evaluations often provide cross-sectional or short-term evidence, yielding average estimates that mask heterogeneity across socioeconomic and demographic subgroups. Moreover, conventional approaches often overlook the dynamic feedback processes underlying dietary adaptation, industry reformulation, and food substitution processes that fundamentally shape the effectiveness of nutrition policies at the population level.

To address these methodological limitations, researchers have increasingly adopted microsimulation modeling to evaluate nutrition and obesity-related policies. Microsimulation operates at the individual level, allowing dietary behaviors, risk factors, and policy interventions to evolve over time. By capturing population heterogeneity, behavioral adaptation, and long-term cumulative effects, microsimulation provides a more realistic assessment of policy impact than static or aggregate models. In recent years, microsimulation has gained recognition as an evidence-based tool for researching obesity and nutrition-related public policies, projecting the long-term effects of dietary, fiscal, and environmental interventions on obesity trends, chronic disease burden, and health equity [6,7].

Despite a growing number of simulation-based evaluations of nutrition and obesity policies, only a limited set of reviews has systematically examined how microsimulation models are used specifically for these interventions. Some earlier reviews have combined microsimulation with other modeling approaches such as system dynamics and agent-based models [7,8]. Others focused narrowly on single policy domains, particularly sugar-sweetened beverage taxation [9]. Consequently, there is still insufficient synthesis of how microsimulation models are designed, calibrated, validated, and applied across diverse policy mechanisms. A better understanding of these methodological choices is essential to enhance the credibility of simulation-based evidence and its translation into adequate nutrition and obesity-related policy decisions.

This scoping review examines the use of microsimulation methods in evaluating nutrition policies related to obesity. The review focuses on the design and structure of individual-level microsimulation models applied in obesity-related policy interventions, with particular attention to behavioral parameterization and the incorporation of economic and equity analyses. Specifically, it categorizes studies by the structure and scope of nutrition policy interventions, examines how obesity is represented and modeled within microsimulation frameworks, and assesses how equity considerations are addressed in model design and reporting. Through this synthesis, the review aims to advance methodological understanding and inform the development of transparent and policy-relevant microsimulation frameworks for nutrition and obesity policy evaluation.

## 2. Materials and Methods

### 2.1. Search Strategy

This scoping review followed the Preferred Reporting Items for Systematic Reviews and Meta-Analyses (PRISMA) guidelines, and its protocol was prospectively registered in PROSPERO (CRD42024599769). A comprehensive search was conducted in PubMed, Web of Science, the Cochrane Library, Scopus, and EBSCO for English-language publications from database inception through 27 August 2024.

The search strategy was developed around three conceptual domains: microsimulation, obesity, and public policy interventions. Boolean operators and controlled vocabulary terms (MeSH, where applicable) were combined to ensure completeness. In PubMed, the core search string was formulated as: (“microsimulation”) AND (“overweight” OR “obesity” OR “adiposity” OR “body mass index” OR “BMI” OR “body weight” OR “waist circumference” OR “waist-to-hip” OR “adipose tissue”) AND (“policy” OR “program”).

**Figure 1:**
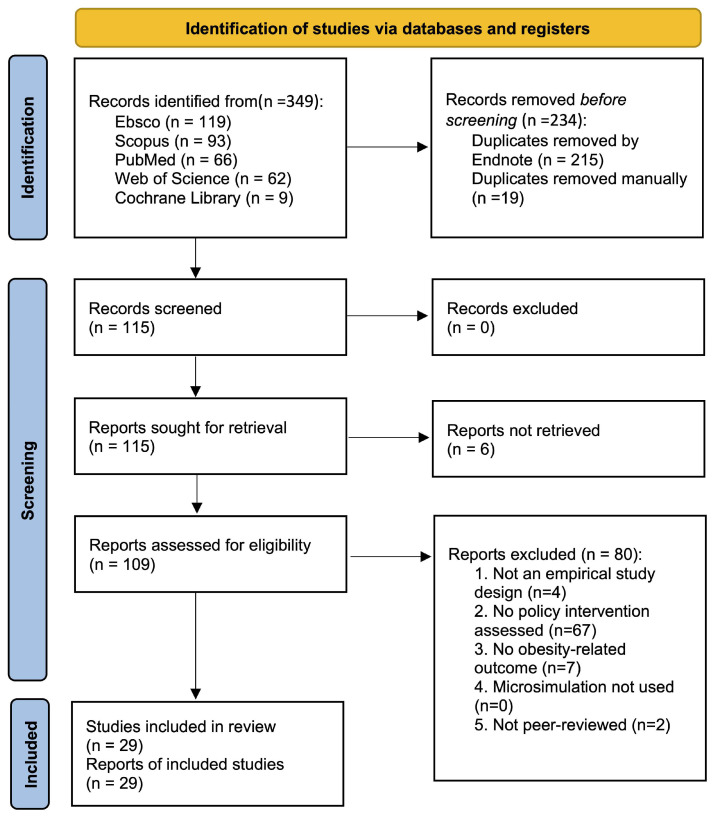
PRISMA flow diagram for the Selection of Obesity Policy Microsimulation Studies.

Search syntax was adapted for each database to account for differences in indexing and search fields. Additionally, the reference lists of all eligible publications and relevant reviews were manually screened to identify any additional studies not captured through the electronic searches. Detailed database-specific search strategies are provided in Figure 1.

### 2.2. Eligibility Criteria

Explicit inclusion and exclusion criteria were established to ensure the selection of empirical and policy-relevant microsimulation studies focusing on obesity-related outcomes.

Studies were included if they met all the following criteria. (1) Study design: empirical analyses using quantitative data and presenting original research findings; (2) Modeling approach: application of a microsimulation model to assess the effects of policy interventions; (3) Policy scope: evaluation of specific public policies implemented at the national, state or regional, or city level that were intended to influence obesity-related outcomes; (4) Outcomes: inclusion of obesity-related indicators such as body mass index (BMI), body weight, or waist-to-hip ratio as primary or secondary outcomes; (5) Publication type: original, peer-reviewed journal articles reporting empirical results; and (6) Language: published in English.

Studies were excluded if they (1) analyzed interventions that were not explicitly policy-based, such as community programs or educational initiatives lacking a formal policy framework; (2) focused exclusively on related behaviors such as diet, physical activity, or general health promotion without reporting obesity-related outcomes; (3) consisted of commentaries, opinion pieces, reviews, theoretical papers, conference abstracts, or methodological reports without empirical data; or (4) were not written in English.

### 2.3. Data Extraction

Data extraction was conducted independently by three reviewers (Y.F., Z.C., and C.W.), and all information was recorded in structured Microsoft Word tables to ensure consistency and traceability. To facilitate cross-referencing throughout this review, each included study was assigned a unique Study ID (S1–S29) based on the order of appearance in Table A1. These identifiers are used to refer to studies within the text and tables to avoid confusion with the numerical citation system. Extracted data included the following elements: modeling domain, components, sub-components, description, and study IDs. To maintain accuracy and completeness, each reviewer cross-checked extracted entries against the original publication, and discrepancies were resolved through discussion and consensus.

### 2.4. Study Quality Assessment

Because no standardized tool exists for evaluating the quality of microsimulation studies on nutrition policies related to obesity, we developed a customized checklist designed to evaluate both the technical quality and the policy context of each included study. The checklist was informed by established evaluation frameworks, including CHEERS 2022, which provides reporting guidance for economic modeling studies, and the NIH Quality Assessment Tools, which provide criteria for evaluating study design and reporting transparency [10,11]. We applied these frameworks to access the design, transparency, and reporting of the included microsimulation studies.

The final instrument comprised 21 criteria assessing model specification and transparency, quality and representativeness of input data, justification and implementation of policy scenarios, internal and external consistency checks, uncertainty and sensitivity analyses, and ethical or practical considerations. Each criterion was rated on a three-point scale (2 = fully met; 1 = partially met; 0 = not met), yielding a total score ranging from 0 to 42 per study (see details in Table A2).

For consistent interpretation, studies scoring ≥ 36 (≥85%) were considered good quality, demonstrating strong methodological rigor and transparent reporting; those scoring 27–35 (≈65–84%) were rated fair quality, reflecting reasonable rigor but some gaps in reporting or validation; and those scoring < 27 (<65%) were deemed poor quality, with several key methodological aspects insufficiently addressed.

Generative AI tools were used only to assist in preliminary data extraction and study quality assessment. All extracted information was independently verified and finalized by the review author (Z.C.).

## 3. Results

### 3.1. Study Selection and Quality Assessment

Figure 1 presents the PRISMA flow diagram summarizing the study selection process. A total of 349 records were identified through database searches, including EBSCO (*n* = 119), Scopus (*n* = 93), PubMed (*n* = 66), Web of Science (*n* = 62), and the Cochrane Library (*n* = 9). After removing duplicates, 115 unique records were screened by title and abstract. Because these records did not provide sufficient information to determine eligibility, all 115 articles were retrieved for full-text review. Based on the predefined inclusion and exclusion criteria, 85 articles were excluded for the following reasons: ineligible study design (*n* = 4), absence of a policy-intervention component (*n* = 67), lack of obesity-related outcomes (*n* = 7), not published in a peer-reviewed journal (*n* = 2), or full text not available (*n* = 6). Two investigators (Y.F. and C.W.) independently conducted the screening with an inter-rater agreement of 82%. Following consensus discussions with the senior investigator (R.A.), 29 studies were included in the final scoping review.

Based on the customized 21-item quality-assessment checklist (see Table A2), 20 studies were rated as good quality, and the remaining 9 studies were rated as fair quality. Overall, the included microsimulation studies demonstrated transparent reporting, a suitable model structure, and adequate validation.

### 3.2. Structure and Scope of Modeled Nutrition Policy Interventions

#### 3.2.1. Policy Intervention Levels

Policy intervention levels reflected the scale at which nutrition policies were designed and implemented. National policies (*n* = 21) were modeled using nationally representative data to estimate population-wide effects on diet and obesity. State/regional policies (*n* = 7) incorporated subnational demographic, dietary, and economic contexts, while local/institutional policies (*n* = 6) were implemented within cities, schools, or workplaces using context-specific data. Several policy interventions operated across more than one administrative level; therefore, the total number of policies exceeded the number of included studies. Among these, the classification of New York City’s beverage-pricing policy examined by Grummon and Golden (2022) [12] required clarification: although the city’s jurisdiction is larger than that of most counties, the policy was legislated and administered at the municipal level and therefore categorized as local.

#### 3.2.2. Policy Settings by Countries

Most identified microsimulation studies were conducted in U.S. policy contexts (*n* = 25; 86%), with the remainder based in Mexico, Germany, the United Kingdom, and Italy (each *n* = 1; 3%). This pattern underscores the predominance of U.S. empirical infrastructures and modeling frameworks in nutrition-policy evaluation, while demonstrating a gradual international expansion of microsimulation applications to obesity-related policy research.

#### 3.2.3. Policy Mechanism and Policy Intent

Across the 29 included microsimulation studies, nutrition-related policies were categorized into five policy mechanisms and six policy intents, reflecting the policy instruments used to shape food environments, dietary behavior, and obesity-related outcomes.

A policy mechanism refers to the instruments through which nutrition policies are implemented across various institutional or regulatory contexts. These included fiscal policies (*n* = 11), which apply taxes, subsidies, or pricing adjustments to influence purchasing behavior on dietary intakes; information and marketing regulations (*n* = 6), which modify how nutrition information is presented to shape consumer awareness; settings-based policies (*n* = 10), which establish nutrition standards in schools, workplaces, or healthcare institutions; food system and assistance program reforms (*n* = 7), which restructure procurement systems or food-assistance benefits such as SNAP and WIC; and clinical and healthcare system policies (*n* = 7), which integrate nutrition counseling or preventive care into healthcare delivery.

Policy intent describes the primary public health objectives pursued within the simulated policy frameworks. Six domains were identified: reducing unhealthy dietary patterns (*n* = 16); promoting healthy dietary patterns (*n* = 5); supporting informed dietary choices (*n* = 5); addressing dietary disparities and food insecurity (*n* = 8); implementing supplementary behavioral interventions for nutrition (*n* = 5); and expanding clinical nutrition interventions (*n* = 6). Among these, supplementary behavioral interventions were distinct in that they encouraged voluntary improvements in diet and physical activity, functioning as behavioral complements to broader structural or fiscal nutrition policies. Collectively, these intents outline the main objectives of nutrition policy design and the behavioral pathways through which modeled interventions influence dietary patterns, energy balance, and obesity outcomes.

Since several studies addressed more than one mechanism or intent, the total counts across categories exceeded the number of included studies.

#### 3.2.4. Behavioral Dose–Response Models

Behavioral dose–response models describe how nutrition policy interventions were translated into measurable changes in food consumption, energy intake, and body weight within microsimulation models. These models specify the behavioral pathways through which fiscal, informational, and behavioral mechanisms influence BMI/obesity and related health outcomes.

The first mechanism, market incentives and consumption adjustment (*n* = 16), captures the economic response to price or income variation induced by fiscal policies. Two subtypes were identified. The price elasticity effect (*n* = 10) quantifies proportional changes in consumption in response to price variation, using own-price elasticity parameters to estimate how consumers adjust purchasing when prices increase or decrease. The substitution and/or income adjustment (*n* = 10) extends this framework by incorporating cross-price or income elasticities, reflecting compensatory shifts toward untaxed or lower-cost alternatives and the broader effect of income redistribution on dietary behavior. Together, these models illustrate how fiscal interventions reshape consumption patterns and energy balance through economic decision-making.

The second mechanism, information exposure and decision change (*n* = 16), explains how information and marketing environments influence consumer food choices. The labeling or information response (*n* = 5) models behavioral change associated with greater visibility and clarity of nutrition information, such as calorie disclosure or front-of-pack labeling, which helps consumers make more informed purchasing decisions. The marketing and/or setting response (*n* = 11) represents changes in behavior following adjustments to the food environment, including bans on unhealthy food advertising or the adoption of institutional nutrition standards. Parameter values for these models were generally informed by experimental or quasi-experimental studies that quantified how exposure to nutrition information or marketing interventions affects consumption patterns.

The third mechanism, behavioral maintenance and compensation over time (*n* = 10), examines how the effects of nutrition policies change as implementation progresses. The compensatory offset (*n* = 9) describes the partial rebound in consumption or energy intake that occurs when individuals adjust to earlier dietary changes, whereas the adherence and/or persistence decay (*n* = 6) term reflects the gradual reduction in participation or compliance over time. Together, these behavioral dynamics demonstrate how policy effects transition from short-term behavioral responses to longer-term maintenance, emphasizing the need to model sustained changes in dietary behavior within microsimulation frameworks.

Overall, the behavioral dose–response mechanisms outlined here synthesize the full range of approaches used across all included microsimulation studies on obesity-related nutrition policies. Collectively, they show how microsimulation models translate policy actions into dietary and health impacts at the population level.

### 3.3. Equity Considerations in Model Design and Reporting

Across the included microsimulation studies, approaches to assessing equity varied considerably, but most incorporated at least one element addressing the distribution of policy exposure or outcomes. For clarity and comparison, four dimensions were used to classify equity-related analyses: differential exposure, equity metrics, subgroup disaggregation, and equity sensitivity analysis (see Table 1). Together, these dimensions describe how models addressed population heterogeneity, measured inequality, analyzed subgroup variations, and evaluated the robustness of equity-related findings.

Differential exposure was one of the most frequently applied components (*n* = 25), capturing variation in how policies reached and affected different socioeconomic and demographic groups. Many studies incorporated stratified parameters by income, education, sex, or race/ethnicity to represent unequal exposure to policy-relevant risks and conditions. This approach enabled models to account for distributional differences in policy impact rather than assuming uniform effects across the population.

Far fewer studies applied formal equity metrics. Only one study used absolute measures, such as the Slope Index of Inequality or the absolute Concentration Index, and none of the studies employed a relative measure based on the Relative Index of Inequality. None conducted equity-adjusted cost-effectiveness analyses, in which efficiency and fairness are evaluated together within the same modeling framework.

Subgroup disaggregation (*n* = 25) analyzed outcomes stratified by socioeconomic and demographic characteristics to examine how policy interventions affected different population groups. This stratified reporting enabled models to identify distributional differences in projected effects and to highlight which subgroups were most likely to benefit from or be left behind by specific nutrition policies.

Equity sensitivity analysis (*n* = 5) assessed the distributional robustness of model results by varying intervention efficacy or reach across socioeconomic and demographic groups. Through these stratified or scenario-based simulations, studies examined how differences in policy uptake or response could influence the equity of projected outcomes.

### 3.4. Modeling Frameworks Across Microsimulation Studies

Across the 29 studies included in this review, a total of 20 distinct microsimulation models were identified. Among these, three models were used most frequently and thus represent the dominant modeling approaches observed in this review. The CHOICES microsimulation model was the most widely applied (*n* = 8), followed by the DOC-M (*n* = 2) and CVD-PREDICT (*n* = 2) models. This review also includes other models such as IMPACT_NCD_, THEMIS, and OAPol, as well as several unnamed microsimulation models that were developed for various policy scenarios. The characteristics of all included microsimulation studies are summarized in Table 2, which details each model’s model design and data inputs, obesity modeling equations, calibration, and sensitivity and uncertainty analysis.

The CHOICES (Childhood Obesity Intervention Cost-Effectiveness Study) model, developed at the Harvard T.H. Chan School of Public Health, simulates population-level BMI trajectories and projects the long-term health and economic impacts of obesity-related interventions such as sugar-sweetened beverage taxes, front-of-pack labeling, and school nutrition standards. Each policy scenario is evaluated against a counterfactual status quo to estimate changes in obesity prevalence, healthcare cost savings, and quality-adjusted life years [20]. The DOC-M (U.S. Diabetes, Obesity, and Cardiovascular Disease Microsimulation) model, developed at the University of Chicago, integrates obesity, diabetes, and cardiovascular disease within a state-transition framework. It models BMI as a continuous, time-varying risk factor that influences the incidence of chronic disease, mortality, and healthcare expenditures, allowing for the assessment of policy effects on both population health and health disparities [21]. The CVD-PREDICT model, developed at the University of California, San Francisco, was designed to assess the impact of dietary and obesity-related factors on cardiometabolic outcomes. It connects changes in caloric intake or nutrient composition to BMI trajectories and subsequent cardiovascular risks through validated epidemiologic equations, providing a physiological link between dietary exposures and chronic disease outcomes [22].

#### 3.4.1. Model Design and Data Inputs

Among the 29 studies included in this review, 27 used dynamic, stochastic, individual-level, state-transition microsimulation models to examine obesity-related processes and outcomes. One study applied a static, deterministic, individual-level model, and another used a dynamic, stochastic, non-Markov design. Although not all studies explicitly modeled continuous body-mass trajectories, each incorporated obesity as a key outcome within its broader policy-evaluation structure. This section reviews how obesity was modeled across studies, focusing on four main aspects: time horizon, starting cohort generation, age group, definition of obesity, and the functional role of obesity within the model.

##### Time Horizon

Most studies (*n* = 25) included at least one nutrition policy intervention with a long-term time horizon (≥10 years, including lifetime) to evaluate the sustained health, economic, and distributional consequences of obesity-related nutrition policies as risk and disease accumulated over time. Six studies included policies with a mid-term horizon (5–10 years) to examine gradual changes in weight status, metabolic risk, or intermediate cost outcomes. At the same time, three studies included policies with a short-term horizon (<5 years) focusing on immediate behavioral responses and early implementation effects.

##### Starting Cohort Generation

Construction of the starting cohort was a key element of the model design, and the included studies applied two main approaches commonly used in microsimulation research. Survey-weighted resampling (*n* = 11) drew individuals directly from nationally representative surveys using sampling weights, stratification, and primary sampling units (PSU). Repeated resampling, typically performed using bootstrap or jackknife methods, ensured that the simulated population preserved the representativeness of the target sample and properly accounted for sampling uncertainty [13].

In contrast, synthetic cohort generation (*n* = 20) created simulated individuals from cross-sectional or multi-source datasets through statistical matching, data imputation, or microsimulation methods. This approach was applied when longitudinal follow-up data were unavailable, allowing researchers to approximate population trajectories and long-term policy effects [23].

The selection of a cohort-generation approach was largely determined by the type and scope of available data. Studies relying on nationally representative datasets, such as NHANES or MEPS, typically drew participants directly from these sources to preserve empirical population structures and sampling design. In contrast, models that integrated information from multiple datasets or required long-term projections beyond observed cohorts constructed synthetic populations to approximate the demographic and behavioral diversity of the target population and to simulate policy effects under hypothetical scenarios.

##### Classification of Study Populations and Obesity Definitions

Among the 29 studies included in this review, 14 studies evaluated nutrition policy interventions that focused solely on adults, 9 studies examined interventions targeting only children and/or adolescents, and 6 studies assessed policies that included both age groups. Adults were defined as individuals aged 18 years and older, whereas children and adolescents were defined as those younger than 19 years. The overlap at ages 18–19 years was resolved by classifying studies as child/adolescent when the modeled population was predominantly under 19 years or when BMI-for-age growth references were applied; otherwise, studies were categorized as adult-focused.

For adults, definitions of obesity were largely consistent across models. They followed the World Health Organization (WHO) adult BMI classification (1995), in which obesity is defined as a BMI of 30 kg/m^2^ or greater [14]. One U.S.-based model also incorporated clinical criteria for pharmacologic eligibility, defining obesity as a BMI of 27 kg/m^2^ or higher in the presence of at least one obesity-related comorbidity, such as hypertension or type 2 diabetes [15,16].

In contrast, definitions for childhood and adolescent obesity varied across countries. The International Obesity Task Force (IOTF) cut-offs provide age- and sex-specific BMI thresholds derived from international reference data, linking child BMI percentiles to the adult cut-offs of 25 kg/m^2^ and 30 kg/m^2^ for overweight and obesity, respectively [17]. In this review, the IOTF standard was applied in one policy setting based in Italy [24]. The CDC 2000 growth charts (ages 2–19 years) define overweight and obesity among U.S. children and adolescents as BMI-for-age ≥ 85th to <95th percentile and ≥95th percentile, respectively, relative to the 2000 CDC reference population [18]. All U.S. models included in this review adopted the ≥95th percentile threshold based on these charts—a reference derived from data collected between 1963 and 1980, when obesity prevalence was substantially lower than today [25,26]. Meanwhile, Mexico’s national standard follows the WHO 2007 Growth Reference for School-aged Children and Adolescents, defining overweight as >+1 SD and obesity as >+2 SD on BMI-for-age, corresponding to adult BMI values of 25 and 30 at 19 years [19,27].

These variations demonstrate that obesity classification systems are based on country-specific growth references and public health surveillance frameworks. Consequently, differences in obesity definitions may influence the comparability of model-based policy results across settings.

#### 3.4.2. Modeling of Obesity in Microsimulation Studies

##### Obesity Modeling Equations

Across the 29 studies, obesity was represented in two main ways within microsimulation frameworks. In 15 studies, obesity was modeled as a direct outcome, with the simulation explicitly updating body weight or BMI over time in response to policy-induced changes in diet, activity, or related determinants. In the remaining 14 studies (38%), obesity functioned as an intermediate outcome, serving as the conduit through which policies affected downstream disease incidence, mortality, and healthcare costs. Building on these representations, four families of equations were used to capture the evolution of obesity, ranging from mechanistic energy-balance systems to empirically calibrated BMI trajectories.

1. Dynamic energy-balance (biophysical) models.
  Models such as the Hall equation and the NIH Body Weight Model describe the body weight as the outcome of a dynamic energy-balance system. Persistent gaps between energy intake and expenditure drive predictable changes in fat and lean mass, modeled through differential equations that adjust energy expenditure as body composition evolves [28,29]. These models capture metabolic adaptation alongside changes in diet composition and physical activity, allowing policy simulations to translate shifts in energy balance into continuous weight or BMI trajectories linked to downstream health and cost outcomes [28,29].
2. Empirical regression BMI-transition models.
  Instead of modeling physiology, these approaches estimate changes in BMI statistically using longitudinal or repeated cross-sectional data. Linear or log-BMI regressions, including GAMLSS specifications, are commonly applied. They can be calibrated directly to survey data and capture subgroup heterogeneity, though at the cost of physiological realism.
3. Pediatric energy-balance growth models. Biophysical models represented by the Hall–Butte equations extend the adult energy-balance models to reflect the metabolic demands of growth in children and/or adolescents. They are typically aligned with WHO (2007) or CDC (2000) growth standards to reflect developmental energy needs [30]. By explicitly modeling the accrual of fat and lean tissue and distinguishing normal growth from excess weight gain, these models generate BMI-for-age trajectories under nutritional, physical activity, or school-based interventions [30].
4. Empirical growth-trajectory models. These models estimate changes in BMI distribution over time using large, nationally representative datasets, emphasizing empirically derived trajectories of weight change rather than physiological mechanisms. In most of the included studies, this approach was implemented within the CHOICES microsimulation model, which utilizes the Ward et al. (2017) quantile BMI growth equations [31] to create hypothetical cohorts and project population-level changes in obesity prevalence under various policy scenarios. In contrast, Study 19 used the Osteoarthritis Policy (OAPol) Model, which represents the progression and treatment of knee osteoarthritis while incorporating the influence of obesity on disease development and quality of life.

Collectively, these four modeling approaches summarize how obesity was represented across the included studies. Dynamic energy-balance models and pediatric energy-balance growth models use similar energy-balance equations to simulate body-weight change, applied, respectively, to adult and child or adolescent populations. In contrast, empirical regression BMI-transition models and empirical growth-trajectory models are data-driven, using observed or estimated BMI trajectories to generate calibrated projections for evaluating the population impact of nutrition policy interventions.

##### Calibration

Across the 29 included studies, four main calibration approaches were identified. Survey-weighted calibration (*n* = 28) was the most common method, aligning model inputs and baseline BMI distributions with nationally representative survey data to preserve population structure and sampling variance. Regression-fit calibration (*n* = 8) estimated BMI or risk-equation parameters directly from cohort or clinical datasets using flexible regression models or standardized epidemiologic risk functions such as the Framingham equations. Cross-cohort calibration (*n* = 1) jointly calibrated model trajectories against multiple cohorts or external risk models to ensure consistency across populations. Finally, external data matching (*n* = 6) evaluated predictive performance by comparing simulated and observed outcomes using quantitative goodness-of-fit metrics such as observed-to-expected (O/E) ratios, root-mean-square error (RMSE), Brier score, or c-statistics.

Overall, these calibration practices illustrate how most included microsimulation studies prioritized population representativeness, while a smaller number incorporated regression-based or multi-cohort calibration combined with external validation to strengthen predictive performance.

#### 3.4.3. Sensitivity and Uncertainty Analyses

Sensitivity and uncertainty analyses were conducted in most included microsimulation models to evaluate the robustness of projected obesity-related policy outcomes. Two major analytical approaches were identified: Probabilistic Sensitivity Analysis and Deterministic (One-way) Sensitivity Analysis.

Probabilistic Sensitivity Analysis (PSA) was reported in 24 of the 29 studies. These models quantified parameter uncertainty through Monte Carlo or second-order simulations, assigning probability distributions to key inputs such as intervention effects, BMI transitions, and metabolic coefficients. Results were presented as 95% uncertainty or confidence intervals for BMI or obesity prevalence outcomes. The increasing adoption of PSA across recent studies indicates a more systematic effort to quantify parameter uncertainty and to improve the interpretability of simulated policy effects.

Deterministic or one-way sensitivity analyses were conducted in nearly all studies, allowing researchers to test how alternative modeling assumptions influenced projected policy effects. Rather than adhering to a single protocol, studies applied a diverse set of scenarios reflecting different conceptual or implementation uncertainties. The most frequently examined dimension was intervention effect size (14 studies), in which the assumed magnitude of behavioral or physiological response was systematically increased or decreased to evaluate the robustness of predicted BMI or energy-intake changes. Several studies assessed caloric compensation (*n* = 5), adjusting for varying degrees of post-intervention energy recovery or substitution, while others explored policy coverage and compliance (*n* = 10), modifying participation rates or implementation intensity to approximate real-world variation. A comparable number of models tested assumptions about the duration and sustainability of BMI effects (*n* = 8), for instance, shortening or extending the period over which weight changes were maintained before partial rebound. Analyses of policy design parameters (*n* = 10), including excise-tax rate, price pass-through, or lag between policy enactment and behavioral response, were also common, capturing uncertainty in fiscal and information marketing mechanisms. Finally, some studies incorporated dietary replacement scenarios (*n* = 5), examining how substituting targeted foods or beverages with untaxed or alternative items altered overall caloric trajectories.

Taken together, these deterministic analyses reveal how model outcomes depend on both parameter and structural assumptions rather than on statistical uncertainty alone. By varying behavioral responses, implementation reach, and policy design, the studies provided insight into which components most strongly drive predicted intervention effects and where empirical evidence remains limited.

## 4. Discussion

### 4.1. Policy Settings and Coverage of Modeled Nutrition Policies

The geographic and institutional settings of modeled nutrition policies reveal both the strengths and limitations of current microsimulation research. Most studies were conducted in high-income contexts, particularly the United States, where 25 of the 29 included studies were based, followed by one study each from the United Kingdom, Germany, Italy, and Mexico. This concentration largely reflects the availability of high-quality dietary surveillance systems (e.g., NHANES, MEPS, HSE) and well-established infrastructures for linking nutrition, health, and economic data. In contrast, the scarcity of models from developing countries highlights a critical data and capacity gap. As obesity prevalence is accelerating in Latin America, Asia, and Africa, the absence of localized microsimulation evidence limits policymakers’ ability to assess the potential impact and tradeoffs of nutrition policies in diverse settings.

Moreover, the range of modeled policy mechanisms highlights the complexity of real-world nutrition policy environments. Fiscal and regulatory approaches, including taxes, subsidies, and marketing restrictions, were the most commonly simulated, while broader system-level reforms such as SNAP or WIC restructuring and procurement standards, along with clinical and behavioral nutrition programs, appeared far less frequently. Taken together, these patterns suggest that existing microsimulation research has largely focused on policies that are easier to model and quantify, whereas interventions requiring institutional coordination or behavioral change remain less explored. Paying closer attention to feasibility, cross-sector coordination, and enforcement mechanisms would make future simulations more informative for real-world implementation. It will also be important to expand modeling beyond high-income settings and to include a broader range of policy instruments and contextual parameters to improve the generalizability and real-world applicability.

### 4.2. Cost-Effectiveness Considerations and Limitations

Several of the included microsimulation studies reported cost-effectiveness outcomes; however, systematic comparison of cost-effectiveness results across studies is methodologically challenging. The reviewed studies varied substantially in their time horizons, ranging from short-term implementation periods to long-term or lifetime analyses. Because both costs and effects change over time, cost-effectiveness estimates depend on the selected time horizon.

In addition, studies adopted different analytic perspectives, including but not limited to healthcare, governmental, and societal perspectives, which led to structurally different cost-effectiveness estimates that are not directly comparable. These challenges are further compounded by the absence of a single globally accepted willingness-to-pay (WTP) threshold, as thresholds are defined and applied differently across countries. Given the multinational scope of this review, these sources of heterogeneity substantially limit cross-study synthesis of ICERs. Future microsimulation studies could place greater emphasis on cost-effectiveness analysis and provide more explicit discussion of willingness-to-pay thresholds and related decision criteria across different policy and country contexts, thereby improving the interpretability and policy relevance of cost-effectiveness outcomes.

### 4.3. Equity Evaluation Framework in Obesity-Related Nutrition Policy Models

Equity considerations have emerged as an important but still underdeveloped perspective in the simulation modeling of obesity-related nutrition policies. While recognition of distributional concerns has grown in public-health research, the efforts of equity analysis within microsimulation frameworks remain limited and inconsistent. Only a small number of studies applied formal methods to evaluate how policy interventions affect disparities across population subgroups.

Most models incorporated population heterogeneity by stratifying baseline risks or behavioral parameters according to socioeconomic and demographic factors such as income, education, sex, and race/ethnicity. These stratifications improved population representativeness but were seldom designed to measure inequality directly. In most cases, heterogeneity functioned as an input assumption rather than as a policy outcome, restricting the capacity to assess distributional effects systematically.

From the included studies, the most common strategy was differential exposure, in which variations in baseline risk, intervention coverage, or effect size were modeled across population subgroups to reflect socioeconomic and demographic disparities. A number of studies presented distributional results, stratifying health and economic outcomes by socioeconomic and demographic characteristics to capture heterogeneity in policy impact. Although these practices enhance transparency, they do not allow formal quantification or comparison of inequities between groups. Only a few studies applied quantitative measures such as the Slope Index of Inequality or Concentration Index, and none incorporated distributional or equity-adjusted cost-effectiveness analyses that explicitly integrate equity into conventional efficiency evaluations. Based on the results from the 29 included studies, it can be inferred that the current modeling literature still lacks standardized methodologies to systematically evaluate the distributional impact of nutrition policies.

Only a small number of studies incorporated equity-sensitivity analyses into their model design. In these models, intervention effects or coverage levels were allowed to vary across socioeconomic or demographic subgroups to examine the robustness of projected outcomes from a distributional rather than aggregate perspective. Although still uncommon, this represents a notable methodological advance, as it extends conventional parameter-uncertainty analysis to the equity dimension. Embedding such analyses more systematically in future microsimulation model frameworks would help identify circumstances in which interventions that appear cost-effective overall may nonetheless contribute to widening disparities across different population groups.

Given the persistent disparities in diet quality, obesity prevalence, and food security, establishing a coherent and reproducible framework for equity evaluation has become a critical research priority. Future studies should move beyond descriptive subgroup analyses and routinely incorporate quantitative inequality metrics together with distributional cost-effectiveness analysis as part of model evaluation. Integrating these approaches within microsimulation models would enable a more comprehensive assessment of both aggregate and distributional effects of nutrition policies, thereby providing a stronger empirical foundation for designing interventions that promote not only cost-effectiveness but also equity in population health outcomes.

### 4.4. Time Horizon, Methodological Diversity, Data Transparency, and Policy Feasibility

#### 4.4.1. Time Horizon

An important methodological consideration in microsimulation modeling is that the choice of time horizon should be evaluated in relation to how individual BMI trajectories are specified. Longer simulation horizons place greater demands on the credibility of long-term BMI projections and the representation of subgroup heterogeneity, both of which are central to cost-effectiveness and equity analyses. With longer time horizons, model results depend more heavily on how individual BMI trajectories are specified and extrapolated over time, reflecting both parameter uncertainty and structural uncertainty. When the reliability of long-term BMI projections is uncertain, particularly with respect to differences across socioeconomic and demographic subgroups, shorter or intermediate time horizons may be more appropriate for generating explainable and policy-relevant estimates.

#### 4.4.2. Methodological Diversity for Obesity Modeling

From a methodological perspective, the included microsimulation studies evaluating obesity-related nutrition policies reflected substantial heterogeneity in their modeling frameworks. Broadly, four major types of obesity modeling methods can be identified: Dynamic Energy-Balance Models, Empirical Regression-Based BMI Transition Models, Pediatric Energy-Balance Growth Models, and Empirical BMI Trajectory Models.

Dynamic energy-balance models employ differential equations to describe the dynamic relationship between energy intake and expenditure. Representative examples include the energy-balance equations developed by Hall and colleagues and the NIH Body Weight Model. These physiologically grounded frameworks capture metabolic feedback and adaptive responses to changes in diet or physical activity, enabling realistic long-term simulations of body-weight dynamics [29,32]. However, their broader application in population or policy analyses remains limited, given that such mechanistic models depend on detailed physiological parameters and individual-level metabolic data that are often unavailable or difficult to generalize across heterogeneous populations [6].

Empirical regression-based BMI transition models, by contrast, rely on longitudinal or clinical data to estimate individual BMI changes using regression or risk equations, emphasizing statistical associations rather than physiological mechanisms. These models are computationally efficient and structurally simple, and are therefore widely used to quantify policy impacts. Pediatric energy-balance growth models, exemplified by the Hall–Butte equations of childhood energy balance [30], extend the conventional energy-balance framework by incorporating rules for energy allocation that vary by age, sex, and developmental stage, thereby enabling the simulation of dietary or physical-activity interventions in children and adolescents. Empirical BMI trajectory models, as widely applied in the CHOICES framework, reconstruct population-level BMI distributions and temporal trends using nationally representative survey and census data. These empirically calibrated models are computationally efficient and scalable, making them well-suited for national or regional policy scenario analyses [33,34].

Future modeling efforts could move beyond the current divide between physiologically based and empirically driven approaches. Dynamic models offer biological realism by representing metabolic feedback and energy adaptation, whereas empirical models provide flexibility for population-level projection and policy evaluation. Integrating these perspectives through hybrid modeling could combine the mechanistic precision of energy-balance equations with the scalability of empirically calibrated frameworks, allowing simulations that are both physiologically grounded and applicable to diverse policy contexts.

#### 4.4.3. Data Reliability and Missing Data Handling

Data quality and the treatment of missing values are critical to the credibility of microsimulation results. Our review indicates that transparency in how missing data are handled remains limited across most studies. Some mention using imputation and/or removing rows with missing observations, while others focus on maintaining sample representativeness through survey weighting or calibration during model initialization. When missingness is not properly addressed, bias can occur—for instance, if dietary data are disproportionately absent among low-income groups, simulations may underestimate true health disparities. Given the potential implications for policy interpretation, future research should clearly document data-cleaning and imputation procedures and conduct sensitivity analyses comparing complete and imputed datasets to strengthen model robustness, reproducibility, and policy relevance.

#### 4.4.4. Policy Feasibility and Public Acceptability

Implementation feasibility and public acceptability have received relatively little attention in microsimulation studies of nutrition policy. Most models assess cost-effectiveness without fully considering the administrative and behavioral conditions that determine whether a policy can be implemented and sustained. Effective policy implementations depend not only on technical design but also on administrative capacity, institutional coordination, and the responsiveness of stakeholders. Taxation policies, for instance, hinge on the ability of governments to collect and monitor revenues, enforce compliance, and ensure that price changes are appropriately passed through to consumers. In the CHOICES-based California simulation by Lee et al. (2024) [35], implementation costs were explicitly modeled, and sensitivity analyses varied assumptions about pass-through rates, linking fiscal design with the realities of policy administration and implementation. Beyond taxation, interventions such as labeling or marketing restrictions depend on industry cooperation and public understanding to influence behavior as intended. Future modeling work should better account for the administrative, behavioral, and political factors that influence how nutrition policies are implemented. Drawing on qualitative and institutional evidence can help understand how policies function in real-world settings once adopted. Integrating these perspectives would make microsimulation studies more grounded, informative, and useful for policy decision-making.

### 4.5. Practical Implications and Dissemination and Implementation of Microsimulation Models

Building on the discussion of feasibility and real-world implementation, this review also considers how microsimulation models are used in applied nutrition policy analysis. Across the literature, microsimulation models differ in their design and use, particularly in whether outputs are intended primarily for analytic, scenario-based evaluation or for more direct engagement in policy discussion and planning.

Among the models identified, the CHOICES microsimulation framework provides an example of a model developed with particular attention to dissemination and implementation, in which cost-effectiveness and equity outputs are structured to inform policy analysis by state and local public health agencies. Other microsimulation models included in this review, such as IMPACTNCD, DOC-M, and the Microsimulation for Income and Child Health (MICH) model, are primarily used for policy-oriented, scenario-based evaluation of long-term population health, economic, and equity outcomes. Even when not formally embedded in decision-making institutions, these models contribute to policy-relevant evidence by clarifying expected trade-offs across alternative policy scenarios.

In settings where long-term population follow-up data are limited, microsimulation provides a practical basis for ongoing, model-based policy assessment. Rather than being used only for one-time ex ante evaluation, models can be updated as new surveillance, administrative, or program data become available. Through recalibration and repeated simulation, assumptions related to baseline population risk, policy uptake, and behavioral response can be revised, allowing expected policy impacts to be reassessed as implementation conditions change over time.

Improving the practical use of microsimulation in nutrition policy analysis will depend on transparent model documentation, clear reporting of assumptions and uncertainty, and sustained engagement between modelers and policy stakeholders. When combined with empirical monitoring and qualitative evidence, microsimulation can support iterative learning and adjustment, strengthening its role in applied nutrition policy evaluation.

### 4.6. Practical Implications and Dissemination and Implementation of Microsimulation Models

#### 4.6.1. Limitations Related to Causal Evidence and Quality Assessment

This review has several methodological limitations. Although most included studies described a conceptual pathway linking policy interventions to behavior change, BMI trajectories, and subsequent health outcomes, the empirical strength of evidence supporting individual steps in this pathway may vary across studies. Our quality assessment was designed to capture whether such a causal or theoretical structure was specified, rather than to compare the strength of evidence supporting each step (e.g., policy implementation, behavioral response, or BMI change). As a result, quality scores should be interpreted as indicators of conceptual alignment and reporting completeness rather than as a comparative assessment of causal validity. Future reviews could strengthen evidence evaluation by more explicitly examining the empirical support for each component of the causal pathway.

#### 4.6.2. Limitations Related to the Empirical Basis of Model Parameters

One limitation of this review is that key parameters used in microsimulation models to characterize relationships between policy interventions, behavioral responses, and obesity-related outcomes may be informed by empirical evidence of varying quality, which has implications for how simulation results are interpreted. In obesity-related microsimulation modeling, assumptions about key parameters are often informed by different types of empirical research and study designs.

As an illustration, model parameters may, in principle, be informed by different forms of empirical evidence. For example, parameters describing price–consumption relationships can reflect estimates synthesized from systematic reviews or meta-analyses, or alternatively, results drawn from single empirical studies. Likewise, parameters linking behavioral change to BMI outcomes can be informed by estimates derived from randomized controlled trials, quasi-experimental studies, or observational analyses, which may lead to different levels of confidence in the resulting assumptions.

Accordingly, findings from microsimulation studies should not be interpreted as being determined solely by model structure or technical implementation. Their reliability also depends on the empirical basis used to inform key parameter assumptions. Because this study is a scoping review, we did not seek to formally compare the causal strength of individual parameters or their underlying evidence sources. Rather, by explicitly noting heterogeneity in the evidentiary basis of commonly used model inputs, we aim to guide readers to consider evidence quality when interpreting results from obesity-related microsimulation studies. Future research could build on this review by more systematically examining how different sources of empirical evidence are used to inform key parameters and how such choices influence projected outcomes, thereby strengthening the interpretability and policy relevance of microsimulation modeling.

## 5. Conclusions

This scoping review finds that microsimulation is widely used to evaluate obesity-related nutrition policies and plays an important role in assessing long-term health, economic, and distributional impacts. However, current applications are marked by substantial methodological heterogeneity, particularly in behavioral assumptions, model calibration, and the treatment of equity, with formal equity metrics used only infrequently.

Based on this central finding, future microsimulation research would benefit from greater methodological transparency and standardization, more systematic incorporation of equity-focused measures, and expanded application across diverse policy and geographic contexts. Addressing these priorities would strengthen the comparability and policy relevance of microsimulation evidence for guiding nutrition policy decisions.

## Figures and Tables

**Table 1:** Overview of Policy Scope, Mechanisms, Modeling Approaches, and Equity Analysis in Microsimulation Studies Evaluating Obesity-Related Nutrition Interventions.

Modeling Domain	Component	Sub-Component	Description	Study IDs
Policy Intervention Levels	National	—	Policies implemented at the national scale and modeled using nationally representative data to assess population-wide effects.	S1, S2, S4, S5, S7, S8, S11–15, S17, S20, S21, S23–29
	State/Regional	—	Policies introduced at the state, provincial, or regional level (excluding cities) are modeled with parameters reflecting regional characteristics and baseline differences.	S3, S6, S22, S10, S13, S18,S24
	Local/Institutional	—	Policies implemented within cities, communities, or institutions are modeled using empirical data from local programs or evaluations.	S9, S13, S16, S19, S24, S27
Policy Settings	United States	—	—	S2, S3, S5–9, S11–13, S15–29
	United Kingdom	—	—	S10
	Mexico	—	—	S1
	Italy	—	—	S14
	Germany	—	—	S4
Policy Domain	Policy Mechanism	Fiscal Policies	Use taxes, subsidies, or price adjustments to shift purchasing behavior and reduce demand for unhealthy foods or beverages.	S3, S4, S9, S13, S14, S22, S23, S25, S27, S28, S29
		Information and Marketing Regulations	Modify how nutrition and marketing information is presented to shape consumer awareness and food choices.	S8, S10, S17, S20, S27, S28
		Setting-Based Policies	Establish food and nutrition standards within schools, workplaces, or other institutions to improve the eating environment.	S1, S6, S8, S13, S15, S16, S21, S24, S27, S28
		Food System and Assistance Program Reforms	Reform food assistance or procurement systems to improve access to and quality of healthy foods.	S2, S11, S13, S18, S22, S23, S29
		Clinical and Healthcare System Policies	Integrate nutrition counseling, medical treatment, or preventive care into healthcare delivery to address diet-related risks.	S5, S7, S12, S13, S19, S26, S27
	Policy Intent	Reduce Unhealthy Dietary Patterns	Limit consumption of high-sugar, high-fat, or high-salt products through fiscal, regulatory, or environmental controls.	S1, S3, S4, S8–11, S13, S15, S16, S20, S22, S25, S27, S28, S29
		Promote Healthy Dietary Patterns	Increase access to and affordability of nutritious foods such as fruits, vegetables, and whole grains.	S2, S5, S23, S27, S29
		Support Informed Dietary Choices	Improve transparency and empower consumers by providing clear, accurate, and accessible nutrition information.	S6, S17, S20, S27–28
		Address Dietary Disparities and Food Insecurity	Reduce inequities in healthy food access and alleviate food insecurity in low-income or vulnerable groups.	S2, S5, S11, S13, S14, S22, S23, S29
		Supplementary Behavioral Interventions for Nutrition	Encourage voluntary behavior changes in diet and physical activity to complement structural policies.	S13, S18, S24, S27–28
		Clinical Nutrition Interventions	Expand clinical programs that deliver dietary, pharmacologic, or therapeutic care to prevent or manage nutrition-related diseases.	S5, S7, S12, S19, S26, S27
Behavioral Dose–Response	Market Incentives And Consumption Adjustment	Price Elasticity Effect	ΔC = εₚ × (ΔP/P_0_); Consumption change (ΔC) in response to price variation (ΔP); εₚ = own-price elasticity.	S3, S4, S9, S11, S22–23, S25, S27–29
		Substitution or Income Adjustment	ΔCᵢ = Σⱼ εᵢⱼ × (ΔPⱼ/Pⱼ); Cross-category consumption shift from substitution or income effects	S3, S4, S9, S11, S22, S23, S25, S27-S29
	Information Exposure—Decision Change	Labeling or Information Response	ΔI = α × ΔInfo; Behavioral shift linked to increased information salience (ΔInfo)	S6, S8, S17, S20, S27
		Marketing or Setting Response	ΔE = β × ΔM; Change in behavior (ΔE) following reduction or modification of marketing exposure (ΔM); β derived from policy evaluations such as advertising bans or school-setting standards.	S1, S2, S8, S10, S13, S15, S16, S20, S24, S27, S28
	Behavioral Maintenance and Compensation Over Time	Compensatory Offset	ΔNet = ΔGross × (1 − θ); Net effect after partial behavioral compensation; θ = fraction of offset. Applied in caloric-compensation or substitution modeling.	S1, S4, S8–11, S16, S17, S20
		Adherence/Persistence Decay	$Bₜ\;=\;B_{0}\;\times \;e^{-\lambda t}$; Decline in behavioral adherence with time; λ = attrition rate. Used for participation-dependent programs.	S12, S18, S19, S26–28
Equity Evaluation Framework	Differential Exposure	—	Stratified baseline risks and behaviors across socioeconomic and demographic variables to reflect unequal exposure to policy-relevant risk factors and conditions.	S1–18, S20, S21, S23, S25, S27–29
	Equity Metric	Absolute Inequality Measures	Quantify absolute gaps in health outcomes across socioeconomic and demographic groups. Common metrics include Slope Index of Inequality (SII)**,** Concentration Index (absolute version), and Population-attributable risk differences.	S10
		Relative Inequality Measures	Assess relative disparities in outcomes between the most and least advantaged groups. Common metrics include the Relative Index of Inequality (RII), Concentration Index (relative version), and top-to-bottom outcome ratios.	—
		Equity-Adjusted Cost-Effectiveness	Integrate efficiency and fairness by evaluating how costs and benefits vary across population subgroups. Common approaches include Net Monetary Benefit (NMB) or Equally Distributed Equivalent (EDE) frameworks, often stratified by income or IMD quintiles.	—
		Subgroup Disaggregation for Outcome	Report health and economic outcomes by socioeconomic and demographic subgroup, identifying who benefits most without summarizing inequality in a single index.	S1–17, S19, S20, S21, S23, S25–26, S28–29
	Equity Sensitivity Analysis	—	Stratified or scenario-based modeling varying intervention efficacy or reach by sex or socioeconomic status to assess distributional robustness.	S1, S5, S10, S25, S29

**Table 2:** Overview of Microsimulation Model Structures and Parameters Used to Simulate Obesity Outcomes in Nutrition-Related Policy Evaluations.

Modeling Domain	Component	Sub-Component	Description	Study IDs
Model Design	Model Type	Dynamic, stochastic, individual-level, state-transition	These models simulate how individuals move between health states over successive time steps. They incorporate stochastic variation to reflect uncertainty and population heterogeneity, allowing dynamic changes in risk factors and outcomes over time.	S1–24, S26–28
		Static, deterministic, individual-level microsimulation	These models simulate outcomes for a fixed population of individuals over time using predefined equations without probabilistic state transitions to project how continuous variables such as BMI or mortality evolve deterministically in response to policy-driven changes.	S25
		Dynamic, stochastic, individual-level microsimulation model(non-Markov)	This model simulates individual behaviors and outcomes over time using stochastic variation to capture uncertainty and heterogeneity, allowing dynamic changes in diet, BMI, and disease risk across the simulated population.	S29
	Model Framework	Dynamics of Childhood Growth and Obesity (DCGO)	Simulates patterns of growth, energy balance, and weight change in childhood to estimate long-term obesity trajectories and related health outcomes.	S1
		CHOICES microsimulation	Evaluates the population-level and economic impacts of obesity prevention policies using U.S. demographic and behavioral data.	S2, S3, S6, S13, S21, S22, S24, S27
		IMPACTNCD (Germany)	Projects future noncommunicable disease incidence and mortality based on changes in diet, BMI, and metabolic risk factors over time.	S4
		US Diabetes, Obesity, CVD Microsimulation (DOCM)	Integrates metabolic and cardiovascular pathways to estimate how body weight and risk factors jointly affect disease progression and costs.	S5, S7
		SPHR diabetes prevention microsimulation model(adapted for London)	Assesses the potential long-term health and fiscal outcomes of diabetes prevention interventions implemented in local public health systems.	S10
		The Health Economics Medical Innovation Simulation (THEMIS)	Models the lifetime health, productivity, and healthcare costs associated with obesity-related diseases and medical innovation in U.S. populations.	S12
		Microsimulation for Income and Child Health (MICH) model	Examines how household income dynamics and social policy scenarios influence child growth, obesity risk, and health inequalities.	S14
		CVD-PREDICT	Estimates cardiovascular morbidity, mortality, and cost outcomes using evolving metabolic risk profiles that include BMI, blood pressure, and cholesterol.	S15, S17
		Future Americans Model (FAM)	Forecasts future population health, functional status, and healthcare expenditures under alternative obesity and chronic disease scenarios.	S18
		Osteoarthritis Policy (OAPol) model	Represents the progression and treatment of knee osteoarthritis while accounting for the influence of obesity on disease development and quality of life.	S19
		Obesity-Related Behavior (ORB) model	Simulates how changes in children’s diet, physical activity, and policy-driven behavioral environments affect BMI trajectories, obesity prevalence, and disparities across demographic subgroups over time.	S28
		IHS Life Sciences US Diabetes/Obesity Microsimulation model.	Simulates how changes in body weight affect biomarkers, chronic disease progression, and medical expenditures over time in nationally representative U.S. populations.	S26
		Unnamed model	A customized microsimulation framework was developed to project obesity-related health and economic outcomes for a defined population.	S8, S9, S11, S16, S20, S23, S25, S29
	Time Horizon	Short-term (<5 years)	Focuses on immediate program implementation and behavioral responses before measurable population health changes occur.	S1, S19, S14
		Mid-term (5–10 years)	Examines gradual changes in weight status, metabolic risk, and intermediate health or cost outcomes.	S7, S9, S14, S17, S19, S20
		Long-term (≥10 years, including lifetime)	Assesses the sustained health, economic, and distributional consequences of obesity prevention policies as risk and disease accumulate over time.	S2–8, S10–18, S21–29
Data Inputs	Starting Cohort Generation	Survey-weighted resampling	Researchers use sampling weights, stratification, and primary sampling unit (PSU) to perform repeated sampling, typically through bootstrap or jackknife procedures, to ensure that model estimation or simulation maintains population representativeness and appropriately propagates sampling uncertainty [13].	S1, S5, S7, S8, S10, S11, S13, S15–17, S20
		Synthetic cohort generation	Synthetic cohort generation refers to creating a cohort of simulated individuals from cross-sectional or multi-source data through statistical matching, imputation, or microsimulation methods to approximate longitudinal population trajectories when real follow-up data are unavailable.	S2–4, S6, S9, S11, S12, S14, S15, S18, S19, S21–29
	Age Group	Adults only	Adults are defined as individuals aged 18 years and older.	S4, S5, S7–9, S12, S15, S17–20, S25, S26, S29
		Children/adolescents only	Children/adolescents are defined as ages < 19 years; the 18–19 overlap is handled by classifying studies as child/adolescent when the population is predominantly <19 or uses BMI-for-age metrics, otherwise as adult.	S1, S2, S6, S11, S13, S14, S21, S24, S28
		Mixed	/	S3, S10, S16, S22, S23, S27
Obesity Outcome Specification	Definition of Adult Obesity	WHO adult BMI classification (1995)	Defines adult obesity based on body mass index (BMI), with thresholds of ≥25 kg/m^2^ for overweight and ≥30 kg/m^2^ for obesity [14].	S3–5, S7–10, S12, S15–20, S22, S23, S25–27, S29
			Adults with BMI ≥ 27 kg/m^2^ with at least one obesity-associated comorbid condition (e.g., hypertension or type 2 diabetes) are eligible for anti-obesity pharmacotherapy [15,16].	S12
	Definition of Children and Adolescents’ Obesity	International Obesity Task Force (IOTF) BMI cut-offs	Provides age- and sex-specific BMI thresholds for children and adolescents derived from international growth reference data, linking child BMI percentiles to the adult cut-offs of 25 kg/m^2^ and 30 kg/m^2^ for overweight and obesity [17].	S14
		CDC 2000 growth charts (ages 2–19 years)	Classifies overweight and obesity in U.S. children and adolescents based on BMI-for-age percentiles, with overweight defined as ≥85th to <95th percentile and obesity as ≥95th percentile relative to the 2000 CDC reference population [18].	S2, S3, S6, S11, S13, S21, S24, S27, S28, S22
		WHO 2007 Growth Reference for School-Aged Children and Adolescents	WHO defines overweight as >+1 SD and obesity as >+2 SD on BMI-for-age, aligned to adult BMI 25 and 30 at 19 years [19].	S1
	Functional role of obesity in the model	Direct outcome	Obesity is modeled as a direct outcome when the simulation explicitly tracks changes in body weight or BMI over time in response to policy interventions, treating obesity itself as the primary endpoint of interest.	S1–3, S6, S8, S9, S11, S13, S14, S20–22, S25, S27, S28
		Immediate outcome	Obesity is treated as an immediate outcome when it functions as an intermediate health state through which policies influence subsequent disease or cost outcomes within the model.	S4, S5, S7, S10, S12, S15–19, S23–24, S26, S29
Obesity Modeling Equation	Dynamic Energy-Balance (Biophysical) Models	Hall model; NIH Body Weight Model; Dynamic energy-balance differential equations	Mechanistic energy-balance frameworks linking sustained caloric imbalance to adaptive fat- and lean-mass changes over time, forming the physiological core of weight-change modeling.	S8–11, S17, S20, S23, S25, S29
	Empirical-Regression BMI Transition Models	Linear or log-BMI regression; Quantile regression; GAMLSS BMI functions	BMI change is estimated using statistical or semi-parametric regression models based on longitudinal or repeated cross-sectional data, without relying on underlying metabolic equations.	S4, S5, S7, S12, S14–16, S18, S26, S28
	Pediatric Energy-Balance Growth Models	Hall–Butte pediatric energy-balance equations; WHO (2007)/CDC (2000) z-score curves	Biophysical growth models combining metabolic adaptation with age- and sex-specific energy requirements to simulate BMI-for-age trajectories in children and adolescents.	S1, S11
	Empirical Growth-Trajectory Models	Empirically derived BMI trajectories (CHOICES, Ward et al.).	Empirical cohort-fitted growth-curve models projecting BMI percentiles through quantile regression or observed secular trends rather than energy-balance equations.	S2, S3, S6, S13, S19, S22, S24, S27
Core obesity-Modeling Structure	Parameter Calibration	Survey-weighted calibration	Model inputs and baseline BMI distributions aligned with nationally representative survey data to preserve population structure and sampling variance.	S1–17, S19–29
		Regression-fit calibration	Parameters of BMI or risk equations fitted directly to cohort or clinical data using flexible regression methods or standardized epidemiologic risk functions (e.g., Framingham).	S5, S10, S12, S14, S17, S18, S23, S26
		Cross-cohort calibration	Model trajectories are jointly calibrated or validated against multiple cohort datasets or independent risk-model predictions to ensure external consistency.	S6
		External data matching	Predictive performance is evaluated by comparing simulated and observed outcomes using quantitative fit metrics such as O/E ratios, RMSE, Brier score, or c-statistics.	S5, S7, S11, S15, S25, S29
Sensitivity and uncertainty analysis	Probabilistic Sensitivity Analysis (PSA)	—	Uncertainty quantified through Monte Carlo or second-order simulations, assigning probability distributions to intervention effects, BMI transitions, and model parameters. Results reported as 95% uncertainty or confidence intervals for BMI or obesity prevalence outcomes.	S1–11, S13–18, S20–24, S27, S29
	Deterministic/One-way Sensitivity Analysis	Intervention effect size	Tested alternative magnitudes of intervention impact on energy intake or BMI.	S1, S5, S7, S10–13, S16, S18–21, S27–28
		Caloric compensation	Examined the degree to which individuals regain calories or weight through physiological or behavioral compensation (e.g., 0–30% caloric regain).	S1, S8, S9, S17, S20
		Policy coverage and compliance	Varied the proportion of the population reached or compliant with the intervention (e.g., 30–100% participation or implementation coverage).	S1, S2, S7, S9, S11, S15, S21, S23, S24, S28
		Duration and sustainability of BMI effects	Tested alternative assumptions about how long BMI or weight changes persist before partial or full rebound	S10, S11, S18–20, S23, S25–26
		Policy design parameter (tax rate, pass-through, lag time)	Evaluated variation in policy design elements such as excise-tax rate, price pass-through, or the lag between dietary change and BMI response.	S3, S4, S9, S13, S14, S23, S25, S27–29
		Dietary replacement	Assessed how replacing restricted items (e.g., SSBs) with alternative foods or beverages alters total energy intake and BMI trajectories.	S4, S11, S16, S23, S25

Model descriptions were synthesized by the authors to provide a general overview of each model’s structure and use in obesity-related microsimulation studies. The descriptions do not represent verbatim statements from the cited sources.

## Data Availability

This study did not generate any new data.

## Abbreviations

The following abbreviations are used in this manuscript:

DOCM	U.S. Diabetes, Obesity, and Cardiovascular Disease Microsimulation
CHOICES	Childhood Obesity Intervention Cost-Effectiveness Study
CVD-PREDICT	Cardiovascular Disease Policy Model for Risk, Events, Detection, Interventions, Costs, and Trends
SSBs	Sugar-sweetened beverages
SES	Socioeconomic Status
SNAP	Supplemental Nutrition Assistance Program
IOTF	International Obesity Task Force

## Appendix A

**Table A1:** Full search strategy.

PubMed		
No.	Search Terms	Results
1	(microsimulation[tiab] OR “micro-simulation”[tiab] OR “micro simulation”[tiab]) AND (overweight[tiab] OR overweight[mh] OR obesity[tiab] OR obesity[mh] OR obese[tiab] OR adiposity[tiab] OR adiposity[mh] OR adipose[tiab] OR “body mass index”[tiab] OR “body mass index”[mh] OR BMI[tiab] OR “body weight”[tiab] OR “body weight”[mh] OR “waist circumference”[tiab] OR “waist circumference”[mh] OR “waist to hip”[tiab] OR “waist-to-hip”[tiab] OR “waist-hip”[tiab] OR “waist-hip”[mh] OR “waist hip”[tiab] OR “body fat”[tiab] OR “adipose tissue”[mh] OR “excess weight”[tiab]) AND (policy[tiab] OR policy[mh] OR policies[tiab] OR program[tiab] OR program[mh] OR programs[tiab])	66
Web of Science		
No.	Search Terms	Results
1	AB = (microsimulation OR “micro-simulation” OR “micro simulation”) AND AB = (overweight OR obesity OR obese OR adiposity OR adipose OR “body mass index” OR bmi OR “body weight” OR “waist circumference” OR “waist circumference” OR “waist to hip” OR “waist-to-hip” OR “waist-hip” OR “waist hip” OR “body fat” OR “adipose tissue” OR “excess weight”) AND AB= (policy OR policies OR program OR programs)	58
2	TI = (microsimulation OR “micro-simulation” OR “micro simulation”) AND TI = (overweight OR obesity OR obese OR adiposity OR adipose OR “body mass index” OR bmi OR “body weight” OR “waist circumference” OR “waist circumference” OR “waist to hip” OR “waist-to-hip” OR “waist-hip” OR “waist hip” OR “body fat” OR “adipose tissue” OR “excess weight”) AND TI = (policy OR policies OR program OR programs)	4
3	#1 AND #2	62
Cochrane Library		
No.	Search Terms	Results
1	microsimulation OR “micro-simulation” OR “micro simulation” in Title Abstract Keyword	199
2	overweight OR obesity OR obese OR adiposity OR adipose OR “body mass index” OR BMI OR “body weight” OR “waist circumference” OR “waist circumference” OR “waist to hip” OR “waist-to-hip” OR “waist-hip” OR “waist hip” OR “body fat” OR “adipose tissue” OR “excess weight” in Title Abstract Keyword	174,711
3	policy OR policies OR program OR programs in Title Abstract Keyword	190,908
4	MeSH descriptor: [Obesity] explode all trees	21,563
5	MeSH descriptor: [Adiposity] explode all trees	1129
6	MeSH descriptor: [Body Mass Index] explode all trees	14,128
7	MeSH descriptor: [Body Weight] explode all trees	40,767
8	MeSH descriptor: [Waist Circumference] explode all trees	1480
9	MeSH descriptor: [Waist-Hip Ratio] explode all trees	350
10	#2 OR #4 OR #5 OR #6 OR #7 OR #8 OR #9	178,664
11	MeSH descriptor: [Policy] explode all trees	3361
12	MeSH descriptor: [Program] explode all trees	0
13	#3 OR #11 OR #12	192,160
14	#1 AND #10 AND #13	9
Scopus		
No.	Search Terms	Results
1	TITLE-ABS (microsimulation OR “micro-simulation” OR “micro simulation”) AND TITLE-ABS (overweight OR obesity OR obese OR adiposity OR adipose OR “body mass index” OR bmi OR “body weight” OR “waist circumference” OR “waist circumference” OR “waist to hip” OR “waist-to-hip” OR “waist-hip” OR “waist hip” OR “body fat” OR “adipose tissue” OR “excess weight”) AND TITLE-ABS (policy OR policies OR program OR programs)	93
Ebsco		
No.	Search Terms	Results
1	AB (microsimulation OR “micro-simulation” OR “micro simulation”) AND AB (overweight OR obesity OR obese OR adiposity OR adipose OR “body mass index” OR bmi OR “body weight” OR “waist circumference” OR “waist circumference” OR “waist to hip” OR “waist-to-hip” OR “waist-hip” OR “waist hip” OR “body fat” OR “adipose tissue” OR “excess weight”) AND AB (policy OR policies OR program OR programs)	116
2	TI (microsimulation OR “micro-simulation” OR “micro simulation”) AND TI (overweight OR obesity OR obese OR adiposity OR adipose OR “body mass index” OR bmi OR “body weight” OR “waist circumference” OR “waist circumference” OR “waist to hip” OR “waist-to-hip” OR “waist-hip” OR “waist hip” OR “body fat” OR “adipose tissue” OR “excess weight”) AND TI (policy OR policies OR program OR programs)	3
3	#1 AND #2	119

## Appendix B

**Table A2:** Overview of Included Studies and Corresponding Nutrition Policy Interventions (S1–S29).

Study IDs	Author, Year, Reference	Nutrition Policy
S1	Basto-Abreu et al. (2024) [36]	Mexico’s national ban on nonessential energy-dense foods and beverages (NEDFBs) in schools
S2	Kenney et al. (2024) [37]	2009 national revision of the WIC food package
S3	Lee et al. (2024) [35]	A hypothetical statewide $0.02-per-ounce excise tax on sugar-sweetened beverages in California
S4	Emmert-Fees et al. (2023) [38]	National SSB taxation policies in Germany (20% ad valorem tax, an extended tax covering fruit juice, and a tiered levy designed to incentivize product reformulation toward lower sugar content)
S5	Kim et al. (2023) [21]	National Produce Prescription Program
S6	Poole et al. (2023) [39]	School-based BMI report cards (BMI feedback letters to parents/guardians)
S7	Wang et al. (2023) [40]	National Produce Prescription Program
S8	An et al. (2022) [41]	SSB Warning Labels; Restaurant Menu Labeling Regulations
S9	Grummon and Golden (2022) [12]	Minimum Price Law (MPL) for SSBs; Excise Tax on SSBs
S10	Thomas et al. (2022) [42]	Transport for London (TfL) Advertising Restrictions on High Fat, Salt and Sugar (HFSS) Products
S11	Choi et al. (2021) [43]	Restriction on the purchase of SSBs using SNAP benefits
S12	Kabiri et al. (2021) [44]	Nationwide Anti-Obesity Medication Uptake Scenario (hypothetical 100% uptake)
S13	Kenney et al. (2021) [45]	Eliminating the Tax Deductibility of Food and Beverage Advertising to Children; Home Visiting Program to Reduce Television Viewing; WIC Motivational Interviewing to Reduce Television Time; Fit5Kids Child Care Curriculum to Reduce Television Time at Home; Policy to Limit Non-Educational Television in Licensed Early Care and Education (ECE) Settings
S14	Rasella et al. (2021) [24]	Basic Income (yearly): the policy provides a universal €100 yearly transfer to all individuals without eligibility requirements; Basic Income (Monthly): the policy provides a universal €100 monthly transfer to all individuals, simulating a stronger income-support effect. Poverty Reduction (yearly): The policy grants €100 yearly per household member to families with per capita incomes below €500 per month; Poverty Reduction (monthly): The policy grants €100 monthly per household member to families with per capita incomes below €500 per month. New-Borns Benefits (yearly): the policy provides €500 yearly for each child under one year old in households with equivalized income below €500 per month; New-Borns Benefits (monthly): the policy provides €500 monthly for each child under one year old in households with equivalized income below €500 per month. Child Benefit (yearly): the policy provides €500 yearly for each child under five years old in households with an equivalized income below €500 per month; Child Benefit (monthly): the policy provides €500 monthly for each child under five years old in households with an equivalized income below €500 per month.
S15	Shangguan et al. (2021) [46]	National Salt and Sugar Reduction Initiative (NSSRI) Voluntary Sugar Reduction Policy
S16	Basu et al. (2020) [47]	Workplace Ban on SSB Sales
S17	Liu et al. (2020) [48]	U.S. Federal Menu Calorie Labeling Law (National Menu Labeling Policy, Section 4205 of the Affordable Care Act, implemented in 2018)
S18	Russell-Fritch et al. (2020) [49]	Supplemental Nutrition Assistance Program Education (SNAP-Ed)
S19	Smith et al. (2020) [50]	Funding an Intensive Diet and Exercise (D+E) Program for Overweight and Obese Patients with Knee Osteoarthritis (OA)
S20	Grummon et al. (2019) [51]	National SSB health warning policy
S21	Kenney et al. (2019) [52]	Water Jets Installation: installs chilled dispensers on school lunch lines to promote water intake and prevent obesity; Grab a Cup, Fill It Up!: adds cup dispensers and signage near fountains to encourage drinking water; Portable Water Dispensers + Promotion: provides portable water jugs with cups and promotion; Bottle-less Water Coolers + Promotion: installs filtered coolers with promotional activities to increase water consumption.
S22	Long et al. (2019) [53]	SSB Excise Tax; SNAP SSB Restriction Policy
S23	Choi et al. (2017) [54]	Fruit and Vegetable (FV) Purchase Subsidy under SNAP
S24	Cradock et al. (2017) [55]	Active Physical Education (Active PE): A state-level policy requiring that at least 50% of PE class time in public K–8 schools be spent in moderate-to-vigorous physical activity. Active Recess: A district-level voluntary program that increases children’s physical activity during recess through structured play, playground markings, and portable equipment. Active School Day: A district-level policy mandating at least 150 min of physical activity per week during the school day through PE, recess, or classroom activity breaks. Healthy Afterschool: A state-level voluntary recognition program that trains and rewards afterschool programs for adopting healthy eating and physical activity practices. New Afterschool Programs: A federally and state-funded initiative providing free, two-hour afterschool sessions in Title I schools with 80 min of supervised physical activity and academic enrichment. Hip Hop to Health, Jr.: A state regulatory policy requiring early childhood education staff to complete structured physical activity promotion training using the Hip Hop to Health, Jr. curriculum.
S25	Pitt and Bendavid (2017) [56]	Hypothetical increase in the retail price of meat
S26	Chen et al. (2016) [57]	Digital Intensive Behavioral Counseling (IBC) Program—Modeled after the National Diabetes Prevention Program (NDPP)
S27	Gortmaker et al. (2015) [34]	SSB Excise Tax; Elimination of the Tax Deductibility for Advertising Unhealthy Foods to Children; Nutrition Standards for All Foods and Beverages Sold in Schools (“Smart Snacks in School”); Restaurant menu calorie labeling; Nutrition standards for school meals; Early Care and Education (NAP SACC) improvements; Increased access to adolescent bariatric surgery
S28	Kristensen et al. (2014) [58]	Strengthening and expanding federally funded afterschool programs to promote physical activity; A national $0.01 per ounce excise tax on SSB; A ban on fast-food television advertising directed at children aged 12 and under
S29	Basu et al. (2013) [59]	Disincentives for SSBs: SSB Ban, SSB tax; Incentives for Fruits and Vegetables: Produce Subsidy, Produce Reward; General SNAP Benefit Increases: Equal-Budget Increase, “Food Stamp Cycle” Change Scenario

## Appendix C

**Table A3:** Model Quality and Reporting Assessment Framework.

No.	Criterion	Ideal Benchmark (Score = 0–2)
1	Research question clarity and theoretical alignment	Research question is explicitly stated, relevant to obesity or nutrition policy, and conceptually grounded in a causal or theoretical framework linking: policy → diet/behavior → BMI → outcomes.
2	Model choice justification	Model type is clearly justified and appropriate for reflecting policy effects over time and population heterogeneity.
3	Representativeness and heterogeneity of data sources	Data are nationally or regionally representative and capture essential heterogeneity by age, sex, socioeconomic status, race or ethnicity, and region.
4	Data reliability and missing data handling	Data sources are validated and well-documented, with clear discussion of limitations and appropriate handling of missing data or bias.
5	High-quality evidence-based parameters	Intervention effects and key parameters are derived from systematic reviews, meta-analyses, or high-quality empirical estimates with defined uncertainty.
6	Model structure transparency	The model’s components, transition logic, and core equations are clearly described or publicly documented for reproducibility.
7	Calibration and validation	The model is calibrated to observed data and externally validated against independent datasets, with quantitative goodness-of-fit or validation metrics reported.
8	Policy definition and justification	The policy intervention is precisely defined, empirically supported, and realistic in its implementation context.
9	Time horizon adequacy	The simulation horizon extends sufficiently (≥ 10 years) to capture long-term or lifetime policy effects and align with decision-making cycles.
10	Dynamic effects	The model distinguishes short-, medium-, and long-term effects and accounts for potential non-linear or saturation dynamics in BMI or behavior change.
11	Assumptions, transparency, and testing	Key assumptions are explicitly stated, evidence-based, and tested through sensitivity or scenario analyses.
12	Equity analysis	The model examines heterogeneity in impacts across population subgroups (e.g., socioeconomic status, race/ethnicity) and reports equity-related outcomes.
13	Cost-effectiveness comprehensiveness	Cost analysis includes both direct (healthcare) and indirect (societal or productivity) costs, with transparent and realistic cost estimates.
14	Policy-relevant outcomes	Outputs include policy-actionable metrics such as BMI trajectories, obesity prevalence, QALYs, cases prevented, and cost per QALY.
15	Sensitivity analyses	The study conducts probabilistic or deterministic analyses to test how varying key assumptions affect model outcomes.
16	Uncertainty characterization	The study identifies and qualitatively discusses parameter, structural, and scenario uncertainties, explaining how these may influence interpretation and confidence in results.
17	Reproducibility and transparency	The model code or detailed documentation is available or accessible, and all assumptions and limitations are clearly stated.
18	Conflict-of-interest disclosure	Funding sources and potential conflicts of interest are fully disclosed, and analytical independence is maintained.
19	Ethical statement	The study clearly states its ethical approval or exemption status, confirms the use of de-identified or publicly available data, and describes consent or data-handling procedures in accordance with institutional and national ethical standards.
20	Implementation feasibility	Potential barriers, scalability, and administrative considerations for implementing the policy are discussed.
21	Policy acceptability	The study considers the policy’s acceptability to key stakeholders, policymakers, and the public, supported by evidence or contextual reasoning.

Note: Each study was rated from 0–2 for each criterion (0 = not met, 1 = partially met, 2 = fully met); total possible score = 42; Criterion 1 evaluates whether a study specifies a coherent conceptual or theoretical causal pathway linking policy interventions, behavioral change, BMI trajectories, and health outcomes. It is intended to capture the presence of such a framework, rather than to assess or compare the empirical strength of evidence supporting each step of the pathway.

**Table A4:** Model Quality Assessment Scores for Included Studies (Study IDs S1–S29).

Criterion	S1	S2	S3	S4	S5	S6	S7	S8	S9	S10	S11	S12	S13	S14	S15	S16	S17	S18	S19	S20	S21	S22	S23	S24	S25	S26	S27	S28	S29
Research question clarity and theoretical alignment	2	2	2	2	2	2	2	2	2	2	2	2	2	2	2	2	2	2	2	2	2	2	2	2	2	2	2	2	2
Model choice justification	2	2	2	2	2	2	2	2	2	2	2	2	2	2	2	2	2	2	2	2	2	2	2	2	2	2	2	2	2
Representativeness and heterogeneity of data sources	2	2	2	2	2	2	2	2	2	2	2	2	2	2	2	2	2	2	2	2	2	2	2	2	2	2	2	2	2
Data reliability and missing data handling	2	1	1	1	2	2	2	2	1	2	1	1	1	1	2	2	1	1	1	2	1	1	1	1	1	2	2	1	2
High-quality evidence-based parameters	2	2	2	2	2	2	2	2	2	2	2	2	2	1	2	2	2	1	2	2	2	2	2	2	2	2	2	2	2
Model structure transparency	2	2	2	2	2	2	2	2	2	2	2	2	2	2	2	2	2	2	2	2	2	2	2	2	2	2	2	2	2
Calibration and validation	2	1	1	2	2	1	2	1	1	1	2	1	2	1	2	1	2	1	2	2	1	1	2	1	2	1	1	1	2
Policy definition and justification	2	2	2	2	2	2	2	2	2	2	2	2	2	2	2	2	2	2	2	2	2	2	2	2	2	2	2	2	2
Time horizon adequacy	0	2	2	2	2	2	2	2	1	2	2	2	2	1	2	2	2	2	1	1	2	2	2	2	2	2	1	2	2
Dynamic effects	2	1	2	2	2	1	2	2	2	2	2	2	2	2	2	1	2	1	2	1	1	1	2	1	2	2	1	1	2
Assumptions, transparency, and testing	2	2	2	2	2	2	2	2	2	2	2	1	2	2	2	2	2	2	2	2	2	2	2	2	2	2	2	2	2
Equity analysis	2	2	2	1	2	2	2	2	2	2	2	1	1	1	2	2	2	1	0	2	1	1	2	0	2	1	0	2	1
Cost-effectiveness comprehensiveness	0	2	1	2	1	2	2	1	0	2	0	1	2	1	2	2	2	0	1	0	2	2	2	2	0	2	1	1	2
Policy-relevant outcomes	2	2	2	2	2	2	2	2	2	2	2	2	2	2	2	2	2	2	2	2	2	2	2	2	2	2	2	2	2
Sensitivity analyses	2	1	2	2	2	2	2	2	2	2	2	0	2	2	2	2	2	2	2	2	2	2	2	2	2	2	2	1	2
Uncertainty characterization	2	2	2	2	2	2	2	2	2	2	2	1	2	2	2	2	2	2	1	2	2	2	2	2	2	1	2	1	2
Reproducibility and transparency	2	2	1	2	2	2	2	1	1	1	2	1	1	2	2	2	1	2	2	1	1	1	2	1	1	1	1	1	1
Conflict-of-interest disclosure	2	2	2	2	2	2	2	2	2	2	2	2	2	2	2	2	2	2	2	2	2	2	2	2	2	2	2	2	2
Ethical statement	2	2	2	2	2	2	2	2	2	2	2	1	2	2	2	1	2	2	1	2	2	2	2	2	2	2	1	1	2
Implementation feasibility	2	2	2	2	1	1	2	1	1	1	1	1	2	1	2	2	2	1	1	1	2	2	1	2	1	2	2	2	1
Policy acceptability	1	1	1	1	1	1	2	1	1	1	1	1	1	1	2	1	1	1	1	1	2	2	1	1	1	2	2	2	1
Sum Score	37	37	37	39	39	38	42	36	34	38	37	30	38	34	42	38	39	33	33	35	37	37	39	35	36	38	34	34	38
Quality Rating	G	G	G	G	G	G	G	G	F	G	G	F	G	F	G	G	G	F	F	F	G	G	G	F	G	G	F	F	G

Note: Studies scoring ≥ 36 were rated good, 27–35 fair, and < 27 poor quality; study IDs are independent of citation numbers for consistency.
